# Seawater Reverse Osmosis Desalination

**DOI:** 10.3390/membranes11040243

**Published:** 2021-03-29

**Authors:** Masaru Kurihara

**Affiliations:** Toray Industries, Inc., 3-2-1 Sonoyama, Otsu-Shi 520-0842, Shiga, Japan; masaru.kurihara.z9@mail.toray

In finalizing this Special Issue “Seawater Reverse Osmosis Desalination”, I would like to express our sincere appreciation to the authors for their contribution of articles and reviews. Seawater reverse osmosis (SWRO), as an unconventional water resource, such as desalinated water, has become one of the most important and indispensable technologies in desalination. The global market status of SWRO desalination plants has gone through a technology transition, from MSF to RO. SWRO plant capacity has expanded from a small size to “Mega-SWRO”. Furthermore, the price of desalinated water is now affordable.

In these circumstances, all SWRO plant technologies require sustainable SWRO desalination as green desalination for the 21st century.

This Special Issue consists of nine papers: six articles and three reviews. The characteristics of this issue are as follows:

1. All the articles and reviews are practical field data based on the scientific approach and essential solution to the problematic matters;
2. Innovative technologies are introduced to solve the biofouling based on biotechnology and have been expanded as a monitoring system;
3. New hybrid system technologies (PRO) are introduced to reduce the energy consumptions and environmental impact;
4. The total plant operation technologies of SWRO are summarized to demonstrate the reliability of the large plants.

In this Special Issue, Miyakawa et al. [1] presents the subsequent progress of “Mega-ton Water System” project and Kurihara et al. developed six technologies in a research project called “Mega-ton Water System”, under a grant from the Japanese society for the Promotion of Science (JSPS), with the aim of developing the sustainable water treatment core technologies necessary for the 21st century, such as green desalination. Furthermore, the Saline Water Convention Corporation (SWCC) conducted joint research with Hitachi and Toray to verify their technologies at Jubail, Saudi Arabia, and the Arabian Gulf. Saudi Arabia appears to be one of the most severe biofouling areas. This demonstration project verified a low-pressure, high-recovery, two-stage system (LMS), which is able to achieve 20% energy reduction and a no-chlorine/no-Sodium Bisulfite (SBS) dosing process, and a biofouling monitoring method (membrane biofouling formation rate (mBFR)) to lessen chemical cleaning interval due to biofouling.

A biofouling monitoring system using an ATB-based bacterial growth potential is presented by A. Abushaban et al. [2]. This article discussed the SWRO pretreatment of a full-scale plant.

The mBFR of “Mega-ton Water System” and this technology are different, despite the fact that both methods use ATB-based bacteria growth potential.

Dissolved air flotation (DAF) and two-stage dual media filter (DMF) were used as a pretreatment process in this article.

A similar article related to biofouling is presented as well [3]. Measuring biofouling potential in SWRO plants by flow-cytometry-based bacteria growth potential was compared with DAF-DMF, DAF-UF, and DAF-CF. DAF-UF afforded good results as a pretreatment.

Assessing pretreatment efficiencies for particulate, organic, and biological fouling in a full plant was studied in the case of chlorine dosing in a full SWRO desalination plant [4]. In this study, DAF was quite effective at reducing biological fouling.

In this Special Issue, H. Takabatake et al. [5] present a comprehensive review of advanced technologies for the stabilization and high performance of a seawater RO membrane plant. This review presents the basic history of SWRO membrane development, typical type of seawater intake, and pre-treatment, including membrane biofilm formation rate (mBFR). It also includes a biofouling index, RO process, energy recovery device (ERD), and a multi-stage for a high recovery ratio, as well as a hybrid system of MBR/sewage RO/UF/seawater.

Cellulose triacetate (CTA)-based hollow fiber membrane is one of the commercially successful semipermeable membrane materials for SWRO [6]. Due to its reliable and excellent performance, especially for obtaining drinking water from seawater, CTA-HF has been widely adopted in arid regions. Furthermore, a newly developed CTA-HF membrane for brine concentration (BC) application (referred to as BC membrane) was also explained.

As well as the Middle Eastern countries, China is a very large market for RO applications. Likewise, China is quite active in the area of research and development of the membrane and its related processes. In particular, the institute of seawater desalination and multipurpose utilization (ISDU), Ministry of Natural Resources is the center of such development in China.

Dr. G. Ruan et al. [7] summarized the progress and perspectives regarding desalination in China. They described the activities of SWRO and SWRO/MED over the 110,000 m^3^/day plant size.

They are broadening the application scenarios and technical integrations, such as island-based seawater desalination and ship use. In addition, China is quite active in developing unconventional membrane materials such as polytetrafluoroethylene (PTFE) hollow-fiber, and ceramic membranes. ISDU has developed MD, FO and CDI, and FO-MD.

Regarding advanced membrane and membrane processes, Pressure Retarded Osmosis (PRO) is an interesting technology. Typical hollow-fiber and polyamide spiral-wounded (PA-SW) membrane modules are available.

Y. Kakihana et al. reported the comparison of the performance in pilot scale test [8]. The maximum power density per unit membrane area is higher in PA-SW due to the higher water permeability. The coefficient of PA-SW, in contrast, the maximum power density per unit volume of CTA-HF, is higher than PA-SW. The value of CTA-HF increased to 13.61 kw/m^3^ at 1.2 M NaCl.

Kyowakiden Industry Co., Ltd. has been working towards introducing PRO to seawater desalination plants since 2001 and is attracting attention for its ongoing pilot plant with a scale of 460 m^3^/day, using concentrated seawater and treated sewage water [9].

Based on the number of seawater desalination plants in each country and the electricity charges, it has been determined that the introduction of PRO would be reliable as potential renewable energy technology.

## References

[1] Miyakawa H., Maghram Al Shaiae M., Green T.N., Ito Y., Sugawara Y., Onishi M., Fusaoka Y., Farooque Ayumantakath M., Saleh Al Amoudi A. (2021). Reliable Sea Water Ro Operation with High Water Recovery and No-Chlorine/No-Sbs Dosing in Arabian Gulf, Saudi Arabia. Membranes.

[2] Abushaban A., Salinas-Rodriguez S.G., Kapala M., Pastorelli D., Schippers J.C., Mondal S., Goueli S., Kennedy M.D. (2020). Monitoring Biofouling Potential Using ATP-Based Bacterial Growth Potential in SWRO Pre-Treatment of a Full-Scale Plant. Membranes.

[3] Dhakal N., Salinas-Rodriguez S.G., Ampah J., Schippers J.C., Kennedy M.D. (2021). Measuring Biofouling Potential in SWRO Plants with a Flow-Cytometry-Based Bacterial Growth Potential Method. Membranes.

[4] Abushaban A., Salinas-Rodriguez S.G., Pastorelli D., Schippers J.C., Mondal S., Goueli S., Kennedy M.D. (2021). Assessing Pretreatment Effectiveness for Particulate, Organic and Biological Fouling in a Full-Scale SWRO Desalination Plant. Membranes.

[5] Takabatake H., Taniguchi M., Kurihara M. (2021). Advanced Technologies for Stabilization and High Performance of Seawater RO Membrane Desalination Plants. Membranes.

[6] Nakao T., Miura Y., Furuichi K., Yasukawa M. (2021). Cellulose Triacetate (CTA) Hollow-Fiber (HF) Membranes for Sustainable Seawater Desalination: A Review. Membranes.

[7] Ruan G., Wang M., An Z., Xu G., Ge Y., Zhao H. (2021). Progress and Perspectives of Desalination in China. Membranes.

[8] Kakihana Y., Jullok N., Shibuya M., Ikebe Y., Higa M. (2021). Comparison of Pressure-Retarded Osmosis Performance between Pilot-Scale Cellulose Triacetate Hollow-Fiber and Polyamide Spiral-Wound Membrane Modules. Membranes.

[9] Makabe R., Ueyama T., Sakai H., Tanioka A. (2021). Commercial Pressure Retarded Osmosis Systems for Seawater Desalination Plants. Membranes.

